# Inverse Correlation Between Distress and Performance in the Medical Rescuers Against COVID-19 in Wuhan

**DOI:** 10.3389/fpsyt.2021.563533

**Published:** 2021-06-24

**Authors:** Fang Xie, Xue Wang, Yun Zhao, Shi Da Wang, Cong Xue, Xiao Tian Wang, Yu Xin Chen, Ling Jia Qian

**Affiliations:** Department of Stress Medicine, Institute of Basic Medical Sciences, Academy of Military Medical Sciences, Beijing, China

**Keywords:** medical rescuer, COVID-19, stress load, performanc, social support

## Abstract

**Background:** During the COVID-19 pandemic, the Chinese government had transferred many medical rescuers to Wuhan, which provided effective support in disease control. The high-intensity working and mental stress during rescue could induce distress and negatively impact the performance of rescuer afterward.

**Materials and Methods:** To identify the characteristics of stress load and its possible effects on performance, the study surveyed 90 medical rescuers in Wuhan using a mobile phone–based self-rated questionnaire.

**Results:** The results showed an existence of universal but mostly mild distress in rescuers. About 95.6% of the participants reported that they had at least one symptom of distress, whereas, the median scores were <30 (100 as max). Compared with civilian rescuers, a higher proportion of working with immediate virus contact was found in military medical rescuers (*P* = 0.008); however, no statistical differences of stress load were found between civilians and militaries. The rescuers with positive cognition or good psychological preparation were found having lower stress loads than other rescuers. An inverse correlation between the stress load and performance (R = −0.24, *P* = 0.023) and a positive correlation between social support and working performance (R = 0.349, *P* = 0.001) were found in our survey, suggesting the possible negative effects of stress and the beneficial effects of social support on performance.

**Conclusion:** Our study indicated that more attention should be paid to the distress of medical rescuers against COVID-19. Positive cognitions, good psychological preparations, and sufficient social support would be necessary to reduce the distress and improve the performance in COVID-19 rescue.

## Introduction

Since January 2020, a severe outbreak of the coronavirus disease in Wuhan has caused over 80,000 infectors in China (1). In order to provide effective medical support for controlling COVID-19, the Chinese government had mobilized and transferred more than 30,000 medical rescuers to Wuhan from January to April in 2020. These actions with other feasible strategies were proved to be very efficient since the pandemic has already been well-controlled in China, and all medical rescuers have already left Wuhan before May 2020. During the antipandemic rescue, these members of the medical staff have suffered both high-intensity work and high-pressure mental stress, which could lead to a variety of distress injuries and harm the performance of rescuers to some extent (2, 3). The information of distress and performance in medical rescuers is not only important in guiding the psychological intervention for those rescuers with mental problems but also beneficial for the country to modify the pandemic coping strategies (4, 5).

Distress contains various symptoms: scare and anxiety could appear immediately after exposure to a stressor, while later-appearing depression, somatic alterations, and even post-traumatic stress symptoms could last for a long time with profound impact (6, 7). Stress load has been introduced as a concept in stress evaluation and has been used to describe distress or potential distress risk quantitatively; however, a standard method for its calculation is still absent. Physiologists tend to use physical or biochemical parameters to reflect somatic alterations (8), whereas, psychiatrists prefer to evaluate the mental problems *via* the psychological diagnose scale (9, 10). In the present study, we created a self-rated questionnaire to identify the characteristics of stress load in four dimensions (depression, anxiety, scare, and somatic distress). Ninety COVID-19 disease medical rescuers in Wuhan were employed to answer this self-rated questionnaire *via* the mobile app WeChat. The performance of rescue of the Wuhan medical staff was also evaluated in the questionnaire survey.

## Materials and Methods

### Participants

Medical rescuers working in Wuhan, including doctors, nurses, medical administrators, and logistic servers, were recruited to participate in this survey *via* a mobile phone app–based questionnaire from February 27, 2020 to April 20, 2020. The study was approved by the Academic Ethics and Security Committee of Academy of Military Medical Sciences. An anonymous, self-rated questionnaire was established using a SaaS cloud platform called “Kuyidian” (wx.kyd5.cn) and was published on the WeChat Official Accounts for Stress Control. The electronic questionnaire was only pushed *via* WeChat to the individuals with informed consent feedback of “yes.”

### Questionnaire

The questionnaire consists of four parts: basic demographic data, subjective view for rescue, stress load assessment, and self-perceived performance status compared to that before medical rescue.

The demographic data included gender, age, marriage status, occupation, education, military or not, working department, and working time per day for rescue. Two levels of virus contact were divided according to the possibility of exposure to confirmed patients. The immediate contact department includes the fever clinic, the emergency department, the general isolation ward, and the intensive care unit. Social supporting status was also included by inquiring how many social and family support they could receive during the rescue process: <50% (rating as 1), 50–80% (rating as 2), 80–100% (rating as 3), or more than 100% (rating as 4) compared to their support before rescue. Subjective view assessment was designed to know the motive of participants for joining the rescue and whether they had worries about their life/health being threatened or getting COVID-19 infection.

In order to minimize the interference to the normal work of rescuers, we self-designed a very brief stress testing questionnaire (only consisted of 24 items) for stress assessment in four dimensions. The items to depression (five items), anxiety (six items), scare (six items), and somatic alteration (seven items) dimension were selected from the Stress Overload Scale, the Self-Rating Anxiety/Depression Scale, and the Depression Anxiety Stress Scale (9–11) and were modified slightly based on these standardized scales. Each item score ranges from 1 (never) to 4 (always), and the stress load score in each dimension was calculated as a normalized (×100 to make it from 0 to 100) ratio between total scores to possible max scores in the respective dimension. The average among scores in four dimensions was defined as the general stress load score. Experts' content validity index (CVI) of each item and Cronbach's alpha coefficients of the total stress questionnaire and each dimension were calculated for content validity and reliability evaluation. Exploratory factor analysis was administrated for stress questionnaire structure test. Accordingly, three items with a lower factor component (one in depression and two in scare) were deleted, and only 21 items were used for stress load assessment finally.

The self-perceived performance status was determined by asking the participants whether their error increased and whether their capacity of execution, comprehension, and judgment declined compared to those before medical rescue. Each question score was set in four grades from 1 (<50% to original capacity) to 4 (>100% to original capacity). Especially, the error question had reversed the score range from 4 to 1 (less than usual is 4, equal to usual is 3, mild increase is 2, and significant increase is 1). The summation of each question score was calculated as the performance score to evaluate the working performance in rescue.

### Statistical Analysis

All statistical analysis was performed by IBM SPSS Statistics 25.0, and data were described using the median and interquartile range. Considering the non-normal distribution of data, Kruskal–Wallis H test was used for the comparison of stress load and performance score. Chi-square test or Fisher's exact test was used to compare the frequency statistics. The correlation between stress load score, social support rating, and performance score was analyzed by Spearman's coefficients. Data were considered statistically significant when *P* < 0.05.

## Results

### Demographic Characteristics and Subjective View

In total, 90 medical rescuers, including 48 (53.3%) doctors, 8 (8.9%) nurses, 23 (25.6%) medical administrators, and 11 (12.2%) logistic servers, returned valid questionnaires. A total of 33 (36.7%) rescuers worked in an immediate virus contact department. General demographic characteristics, such as gender, age, marriage status, etc., are shown in Table 1. Significant differences of identity (military or not) and working time per day were found between the rescuers with immediate or mediate virus contact, respectively. Compared with civilian rescuers, a higher proportion of working with immediate virus contact was found in military medical rescuers (*P* = 0.008). The rescuers with immediate virus contact have a higher proportion in work overtime (*P* = 0.015). Among all the participants, 34 people (37.8%) accepted the rescue as a task, 33 (36.7%) volunteered for rescue, and 23 (25.5%) strived to create an opportunity to join the rescue. The percentages of rescuers with worry for life being threatened, health being threatened, and self-infection were 46.7, 72.2, and 62.2%, respectively. No significant differences were found between immediate and mediate contact rescuers. Thirty-five rescuers could not obtain enough social support, and their ratio equaled statistically in rescuers with immediate or mediate COVID-19 contact.

**Table 1:** Demographic and characteristics of rescue participants for COVID-19 disease, according to contact severity.

		**Contact**		
**Characteristic**	**All participants (*n* = 90)**	**Immediate (*n* = 33)**	**Mediate (*n* = 57)**	***P*-value**
**Demographic characteristic**				
Female—no. (%)	42 (46.7%)	13 (39.4%)	29 (50.9%)	0.293^a^
Median age range—year	30–39	30–39	30–39	-
Married—no. (%)	61 (67.8%)	25 (75.8%)	36 (63.2%)	0.218^a^
Military participates—no. (%)	15 (16.7%)	10 (30.3%)	5 (8.8%)	0.008^a^,^*^
Working time ≥8 h per day—no. (%)	27 (30%)	15 (45.5%)	12 (21.1%)	0.015^a^,^*^
With social support deficiency—no. (%)	35 (38.9%)	16 (48.5%)	19 (33.3%)	0.155^a^
**Subjective view for rescue**				
Accepted the rescue task—no. (%)	34 (37.8%)	11 (33.3%)	23 (40.4%)	0.508^a^
Voluntary for rescue—no. (%)	33 (36.7%)	16 (48.5%)	17 (29.8%)	0.077^a^
Strived for rescue opportunity—no. (%)	23 (25.5%)	6 (18.2%)	17 (29.8%)	0.222^a^
With worry for life threaten—no. (%)	42 (46.7%)	19 (57.6%)	23 (40.4%)	0.114^a^
With worry for health threaten—no. (%)	65 (72.2%)	23 (69.7%)	42 (73.7%)	0.684^a^
With worry for self-infection—no. (%)	56 (62.2%)	23 (69.7%)	33 (57.9%)	0.266^a^
**Stress load assessment**				
With at least one item score >2—no. (%)	86 (95.6%)	31 (93.9%)	55 (96.5%)	0.622^b^
With at least one depression-item score >2—no. (%)	76 (84.4%)	30 (90.9%)	46 (80.7%)	0.241^b^
With at least one anxiety-item score >2—no. (%)	80 (88.9%)	30 (90.9%)	50 (87.7%)	0.74^b^
With at least one scare-item score >2—no. (%)	70 (77.8%)	27 (81.8%)	43 (75.4%)	0.483^a^
With at least one somatic-item score >2—no. (%)	70 (77.8%)	30 (90.9%)	40 (70.2%)	0.034^b^,^*^
**Performance assessment**				
With error increase—no. (%)	2 (2.2%)	2 (6.1%)	0 (0%)	0.132^b^
With execution decline—no. (%)	33 (36.7%)	13 (39.4%)	20 (35.1%)	0.683^a^
With comprehension decline—no. (%)	45 (50%)	15 (45.5%)	30 (52.6%)	0.512^a^
With judgment decline—no. (%)	29 (32.2%)	11 (33.3%)	18 (31.6%)	0.864^a^

* *P < 0.05.*

a *Chi-square test.*

b *Fisher's exact test*.

### Psychometric Properties for Stress and Performance Questionnaire

Based on the results of factor analysis for the original 24-item stress questionnaire, three items with a lower factor component (one in depression and two in scare) were deleted, and only 21 items were used for stress assessment finally. The scale level CVI average (S-CVI/Ave) of the final stress questionnaire increased from 0.94 (24-item) to 0.97 (21-item). Four factors corresponding to the respective dimension were extracted by factor analysis for the new 21-item stress questionnaire, which could explain 63.33% cumulative variance. Cronbach's alpha coefficients for the 21-item stress questionnaire reached 0.93, whereas, that for each dimension ranged from 0.76 to 0.88 (depression 0.80, anxiety 0.88, scare 0.76, and somatic alterations 0.87). The four-item performance assessing questionnaire confided its S-CVI/Ave (1.00) and the Cronbach's alpha coefficients (0.794) under the same analysis. The cumulative variance contribution rate reached 61.875, which indicated acceptable reliability and validity of this plain performance questionnaire.

### Stress Load Assessment

**Figure 1:**
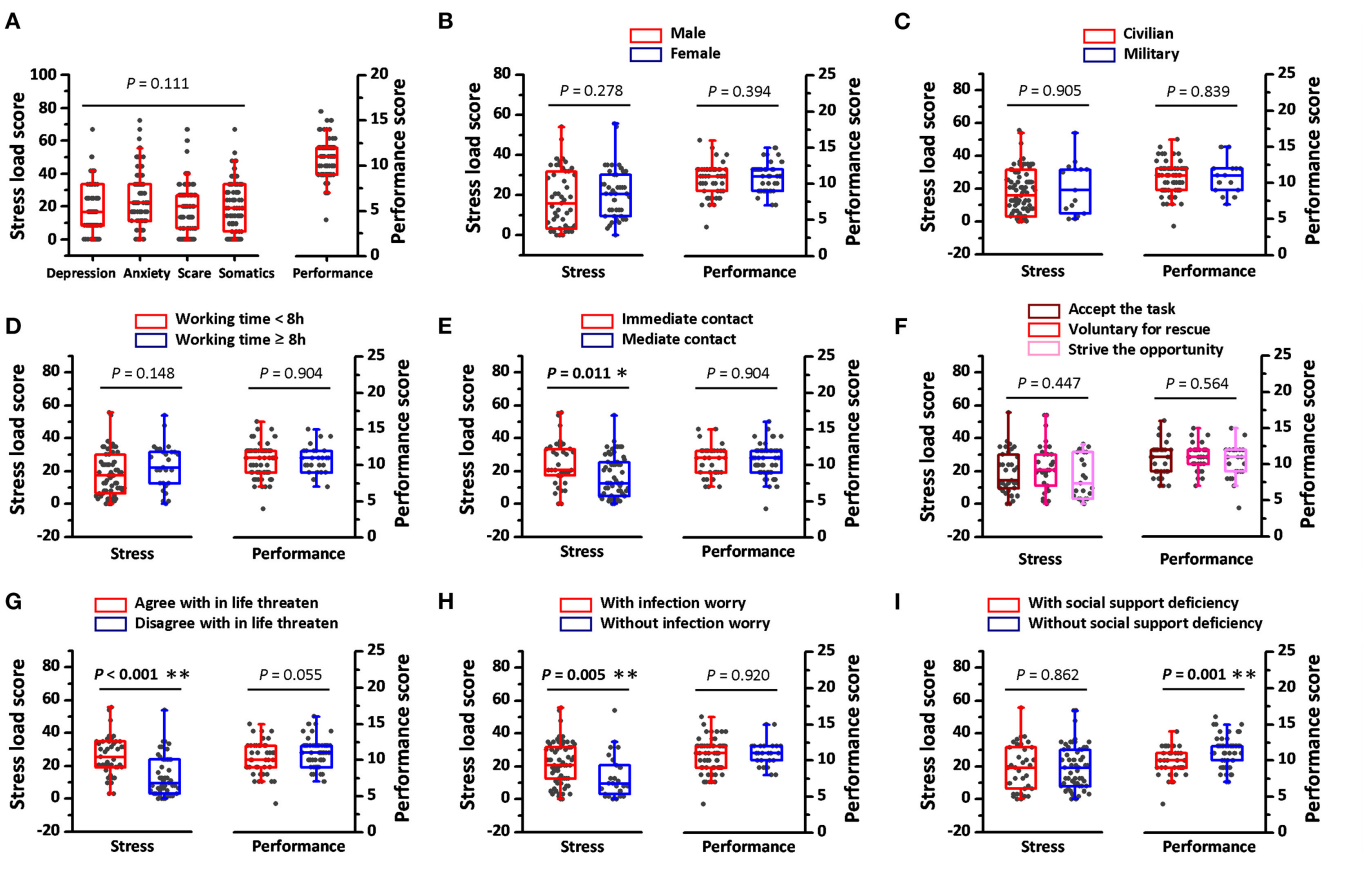
Stress load and working performance assessment in Wuhan medical rescuers. **(A)** The stress load score of all survey participants in depression, anxiety, scare, and somatic alteration dimensions. The stress load score in each dimension was calculated as a normalized (×100 to make it from 0 to 100) ratio between items total scores to possible max scores in the respective dimension. Performance scores, calculated as the summation of four items scores, were also presented to show the performance in all survey participants. **(B–I)** The stress load scores and performance scores in different genders, identities, working time per day, COVID-19 contacts, motives for joining rescue, subjective views, and social supports. The general stress load scores were calculated as the average among the scores in the four dimensions. Social support deficiency was determined as rescuers receiving <80% support compared to that before rescue. Horizontal lines across each box represent medians of the stress load score or the performance score, while heights of each box represent interquartile ranges of stress or performance distribution. *P < 0.05, **P < 0.01.

Although, 95.6% of the participants reported that they had at least one stress symptom (Table 1), the median of the stress load score was maintained at a lower level (Figure 1A), which suggested that the distress in medical rescuers was universal but mostly mild. No statistical differences were identified among the four dimensions in stress load, and according to this, only general stress load scores were shown subsequently. Factors that might affect stress were then verified by comparing the general stress load scores. Gender, military or not, working time per day, motives for joining the rescue, and social supporting did not show statistical impact on the stress load score (Figures 1B–D,F,I), whereas, the immediate COVID-19 contact was found to induce a significant higher stress load score (Figure 1E). Cognition and psychological preparation to rescue could also affect stress loads. Both the worry for possible infection and the self-agreement to having life being threatened lead to higher scores in stress load (Figures 1G,H).

### Performance Assessment

**Figure 2:**
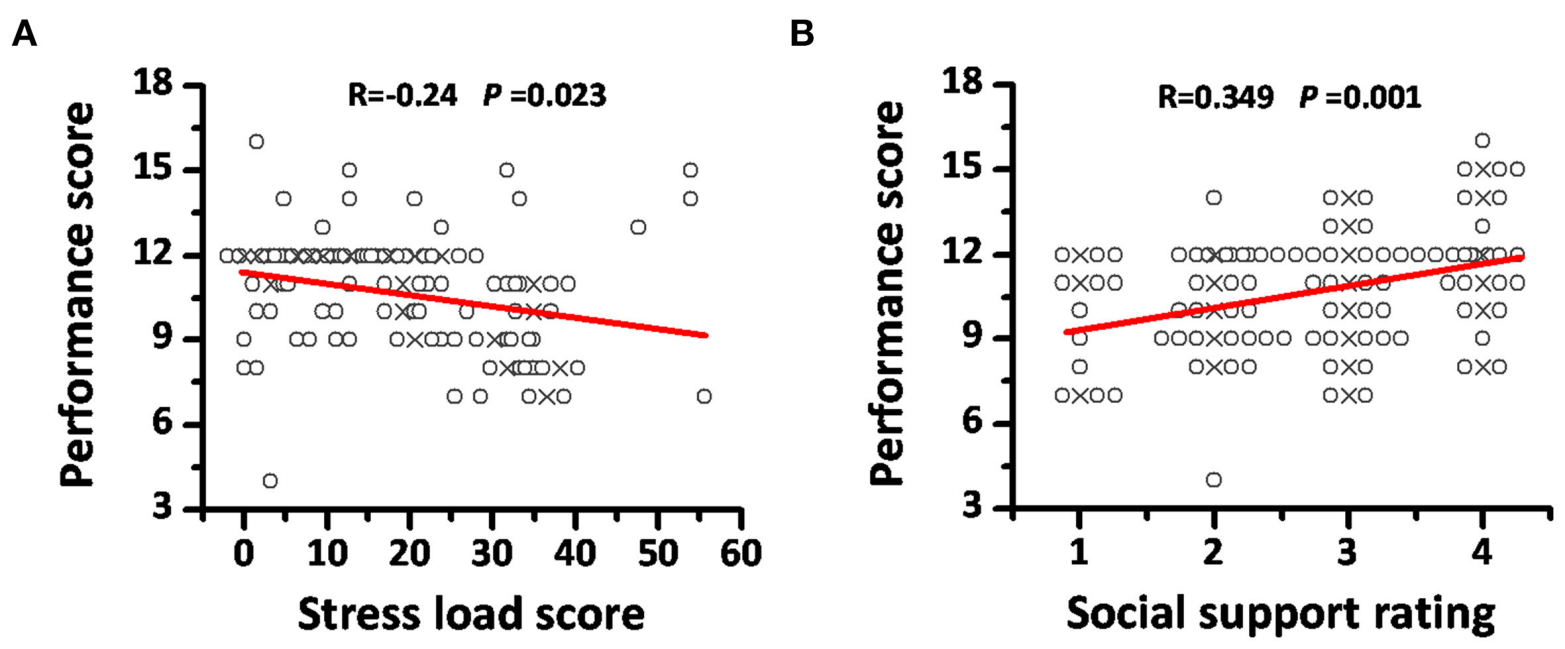
The correlations between the stress load score, social support rating, and the performance score. **(A)** The inverse correlation between the stress load score and the performance score. Stress load scores were calculated as normalized (×100 to make it from 0 to 100) ratios between the item score summation and the possible max score in the 21-item questionnaire. Performance scores were calculated as the summation of four performance items scores. **(B)** The positive correlation between social support rating and the performance scores. Social supporting rating was achieved by inquiring how many social and family support the rescuers could receive during the rescue process: <50% (rating as 1), 50–80% (rating as 2), 80–100% (rating as 3), or more than 100% (rating as 4) compared to their support before rescue. “ × ” means the central position of overlapped points, and overlapped points were plotted as offset.

Only two rescuers in our survey thought their working error increased significantly compared to that before the medical rescue, while many more people reported that they had over 20% performance decline in execution, comprehension, and judgment during the rescue period (Table 1). Performance scores were calculated as the summation of scores of error, execution, comprehension, and judgment, which ranged from 4 to 16 (Figure 1A). Factors that might affect performance were verified, and only sufficient social and family support showed benefits to maintain performance (Figures 1B–I). Spearman's coefficients showed an inverse correlation between the stress load score and working performance (R = −0.24, *P* = 0.023, Figure 2A), and a positive correlation between social support rating and performance (R = 0.349, *P* = 0.001, Figure 2B), suggesting the possible negative effects of stress and the positive effects of social support on performance.

## Discussion

Pandemic outbreak is known as an intensive stressor for medical workers not only because of their direct exposure to the working environment but also owing to the possibility of death of the people around whom they had to face. A notable example would be the severe acute stress reaction of healthcare workers observed during the SARS outbreak in 2003 (7, 12). Stress load assessment is not an effortless task, and the standard method is still absent even today. Most psychiatrists tend to use psychological scales such as the stress overload scale (9), the stress anxiety/depression scale (10) etc., However, members of the hospital staff were found with both physical and psychological stress responses to medical work during the current COVID-19 pandemic (13, 14). Some symptoms of insomnia and myalgia in healthcare workers were thought of as being a result of stress that exacerbates the psychological injury further (3). In the present study, we created a self-rated questionnaire for stress load in both physical and psychological dimensions and identified a universal but mostly mild distress in Wuhan medical rescuers, which is consistent with other studies reporting the subthreshold or mild mental disturbances in more than 70% medical staff members during the COVID-19 pandemic (2). When somatic distress was also included as shown in our study, the proportion of medical rescuers with stress disturbance would be even higher. This mild distress could enhance the rescue motives or immune reaction in medical staff members (2, 15) but would produce some negative effects on their rescue performance. An inverse correlation between the stress load score and working performance was identified in our study, which verified the presumed hazard of stress on rescue performance. To the best of the authors' knowledge, this study is the first one to focus on the association between stress load and performance of medical rescuers in COVID-19 control. On the contrary, good social, and family support was believed to play important roles in health maintenance, especially in the situation of being under stress (16, 17). Consistently, this has also been verified in our present study by the better performance of those rescuers with sufficient social support and the positive correlation between working performance and social support rating.

In stress load assessments, the level of COVID-19 contact was found as the only objective factor impacting stress load. Medical rescuers with immediate virus contact represented higher stress load. Similarly, other surveys also presented the higher risk for suffering depression and anxiety in those medical staff members working with close COVID-19 contact compared to the staff with mediate contact (2, 18, 19). Considering the higher proportion of working with immediate contact in the case of military medical rescuers, the statistical undifferentiated stress load between military and civilian rescuers seems really interesting. The similarity in the working environment between the newly built mobile hospital for COVID-19 patients and the field hospital in military training could possibly account for the better stress resilience of military medical rescuers (20). This implied that the military medical staff members and even some military medicine experience might be beneficial to national-wide COVID-19 control. In contrast with most objective factors, the subjective factors showed more determinative effects on stress load. Our data showed that positive cognition, good psychological preparations, and sufficient social support were helpful for stress load reduction and even performance promotion. This is consistent with most surveys demonstrating that higher trust in infection control predicted less emotional fatigues and anxieties (21–23). These findings highlighted the crucial role of prior preparation and rescue organizing in pandemic control. Clear plans, stable policies, and definitive task arrangements can help medical rescuers focus on key issues in medical operations and reduce their crisis of stress sufferings.

Our study also has certain limitations. First, the measuring instruments used in our study were self-designed especially focusing on the medical rescuers of the Wuhan COVID-19 pandemic, and the validity and reliability of the questionnaire we used were statistically calculated just based on the present survey. Further psychometric properties of these measuring instruments would still be needed and verified in other future studies. Second, most rescuers were too busy and too tired to face the extra burden of survey during the medical rescue against COVID-19. Although, we prolonged our survey to April 20, 2020 (before the evacuation of the medical rescue team from Wuhan), only 90 valid questionnaires were collected. A larger-sized investigation is necessary to verify our results in the future.

## Conclusion

In summary, our study found a possible inverse correlation between universal distress and working performance in the medical rescuers against COVID-19 in Wuhan, which indicated that more attention should be paid to the stress load of medical staff members during the pandemic to maintain their rescue efficiency. Positive cognition, good psychological preparations, and sufficient social support would be helpful to reduce the stress and might improve the performance in COVID-19 rescue.

## Data Availability Statement

The raw data supporting the conclusions of this article will be made available by the authors, without undue reservation.

## Ethics Statement

The studies involving human participants were reviewed and approved by Academic Ethics and Security Committee of Academy of Military Medical Sciences. The patients/participants provided their electronic informed consent to participate in this study.

## Author Contributions

LJQ and FX contributed to the conception and design of the study. FX, XW, YZ, SDW, CX, XTW, and YXC collected and interpreted the data. FX and XW performed the statistical analysis and wrote and revised the manuscript. All authors contributed to manuscript revision and read and approved the submitted version.

## Conflict of Interest

The authors declare that the research was conducted in the absence of any commercial or financial relationships that could be construed as a potential conflict of interest.
